# Efficacy of intermittent pneumatic compression for venous thromboembolism prophylaxis in patients undergoing gynecologic surgery: A systematic review and meta-analysis

**DOI:** 10.18632/oncotarget.13620

**Published:** 2016-11-25

**Authors:** Jian-Ping Feng, Yu-Ting Xiong, Zi-Qi Fan, Li-Jie Yan, Jing-Yun Wang, Ze-Juan Gu

**Affiliations:** ^1^ Department of Anesthesiology, The First Affiliated Hospital of Nanjing Medical University, Nanjing 210029, Jiangsu Province, China; ^2^ Department of Nursing, The Second Affiliated Hospital of Wannan Medical College, Wuhu 241000, Anhui Province, China; ^3^ Department of Nursing, The First Affiliated Hospital of Nanjing Medical University, Nanjing 210029, Jiangsu Province, China

**Keywords:** gynecologic surgery, pulmonary embolism, deep vein thrombosis, intermittent pneumatic compression, heparin

## Abstract

We sought to comprehensively assess the efficacy of Intermittent Pneumatic Compression (IPC) in patients undergoing gynecologic surgery. A computerized literature search was conducted in Pubmed, Embase and Cochrane Library databases. Seven randomized controlled trials involving 1001 participants were included. Compared with control, IPC significantly lowered the deep vein thrombosis (DVT) risk [risk ratio (RR) = 0.33, 95% confidence interval (CI): 0.16 – 0.66]. The incidence of DVT in IPC and drugs group was similar (4.5% versus. 3.99%, RR = 1.19, 95% CI: 0.42 – 3.44). With regards to pulmonary embolism risk, no significant difference was observed in IPC versus control or IPC versus drugs. IPC had a lower postoperative transfusion rate than heparin (RR = 0.53, 95% CI: 0.32 – 0.89), but had a similar transfusion rate in operating room to low molecular weight heparin (RR = 1.06, 95% CI: 0.69 – 1.63). Combined use of IPC and graduated compression stockings (GCS) had a marginally lower risk of DVT than GCS alone (RR = 0.38, 95% CI: 0.14 – 1.03). In summary, IPC is effective in reducing DVT complications in gynecologic surgery. IPC is neither superior nor inferior to pharmacological thromboprophylaxis. However, whether combination of IPC and chemoprophylaxis is more effective than IPC or chemoprophylaxis alone remains unknown in this patient population.

## INTRODUCTION

The incidence of venous thromboembolism (VTE) is more than 100 persons per 100000, and increases with advancing age [1]. The morbidity and mortality of VTE remain high despite the improved prophylaxis, due to various risk factors, such as surgery, trauma, malignancy, hospital, increasing age, lower extremity paresis and so on [2]. Pulmonary embolism (PE) and deep vein thrombosis (DVT) are two main manifestations of symptomatic VTE. There is an increased risk of VTE in perioperative patients. It was reported that there was averagely an incidence of symptomatic VTE of 0.8% within 3 months after operation, and up to 3% in high-risk procedures [3]. Taking into account the fact that approximately 50% of DVT are silent [4], the true frequency of postoperative VTE may even much higher.

Thromboprophylaxis is demonstrated essential in reducing morbidity and mortality of VTE. Low molecular weight heparin (LMWH) is a main recommended thromboprophylaxis for patients undergoing major general surgery. The preventive recommendations for VTE in major gynecologic surgery are similar to that in general surgery [5]. Nonetheless, bleeding risks have precluded the clinical use of antithrombotic drugs [6, 7]. Intermittent pneumatic compression (IPC) is also a recommendation for VTE prophylaxis in patients undergoing major gynecologic surgery for benign disease or malignancy, particularly in patients who are at high risk for major bleeding complications [8, 9]. However, existing guidelines are mainly based on evidence from general surgery, while women undergoing gynecologic surgeryhave additional risk factors for VTE, including oral contraceptive use, estrogen therapy, postpartum period, lithotomy position, malignancy accompanied by high estrogen level, extensive pelvic anatomy and lengthy abdominal and pelvic surgery [10, 11]. Thus evidence generated from studies involving general surgery and men may not be completely suitable for women with gynecologic procedures. Besides, there were few randomized controlled trials (RCT) evaluating the use of IPC in gynecologic surgery at the time of the guidelines publication, and the subsequent studies may further increase our understanding on this topic. Therefore, we conducted this meta-analysis, with aims to comprehensively assess the efficacy of IPC in patients undergoing gynecologic surgery, by means of currently available data.

## RESULTS

### Characteristics of included studies

**Figure 1:**
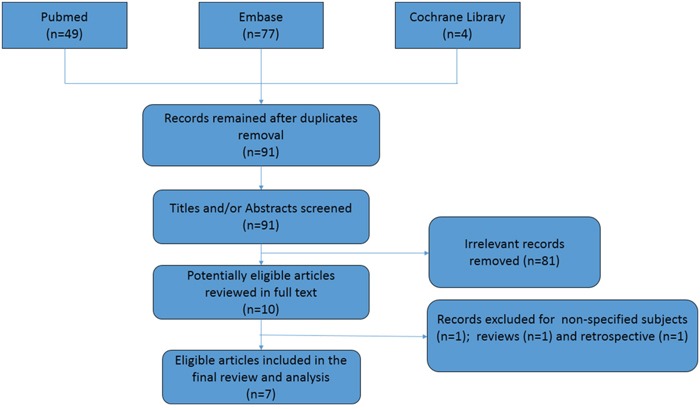
Flow chart of literature search and selection

The literature search and study selection strategy is shown in Figure 1.

A total of 130 citations were initially identified, of which only 7 articles involving 1001 participants were finally included in the analysis [12–18]. Most studies had a low risk of bias (Supplementary Figure 1). Publication bias was shown in Supplementary Figure 2. The messages on treatments group, regimen, sample size and follow up duration were summarized in Table 1. Two studies compared IPC with control [12, 13], of which one used IPC in the perioperative period only (short-dated IPC) [13]. Three trials compared IPC with drugs (heparin or LWMH) [14, 15, 18]. One trial had 3 arms: IPC, LWMH and control [16]. The study by Gao et al. compared a combination of IPC and graduated compression stockings (GCS) with GCS alone, and considered two lower limbs as two samples when calculating DVT rate [17]. As shown in Table 2, a majority studies included patients undergoing major surgery for known or presumed gynecologic malignancies [12–15, 18]. Two studies involved patients undergoing gynecological pelvic surgery with high-risk factors for DVT [16, 17]. However, that 2 study had gynecologic malignancies of 29.4% and 32.4%, respectively.

**Table 1:** Summary of randomized controlled studies on the effect of IPC on DVT and PE

Study/Country	Year	Treatments	Number	Regimen	Endpoints	Follow-up
**Clarke Pearson and Synan et al.America**	1984	IPC	55	Started at the time of induction of anesthesia and maintained for 5 postoperative days.	DVT and/or PE: 7PE: 2	42 days
		Control	52	None	DVT and/or PE: 18PE: 1	42 days
**Clarke Pearson and Creasmann et al.America**	1984	IPC	97	Applied at the time of induction of anesthesia until discharge from the recovery room or 24 hours post operation.	VTE: 18PE: 4	42 days
		Control	97	None	VTE: 12PE: 1	42 days
**Clarke-Pearson and Synan et al.America**	1993	IPC	101	Initiated at the induction of anesthesia and continued for 5 postoperative days.	DVT: 4PE: 0	30 days
		Heparin	107	5000 units at 2 PM, 10 PM, and 6 AM before starting surgery and 5000 units every 8 hours for 7 postoperative days; or until full ambulation or discharge.	DVT: 7PE: 0	30 days
**Maxwell et al.America**	2001	IPC	106	Started at the time of induction of anesthesia and continued for 5 postoperative days.	DVT: 1PE: 0	30 days
		LMWH	105	Received 2500 units subcutaneously before surgery, and then received a daily dose of 5000 units until the 5^th^ day or discharge.	DVT: 2PE: 0	30 days
**Yang et al.China**	2009	IPC	47	Applied at the time of induction of anesthesia until ambulation.	DVT: 4	5 days
		LMWH	48	5000IU the night before operation and continued for 5 days.	DVT: 1	5 days
		Control	48	None	DVT: 10	5 days
**Gao et al.China**	2012	IPC+GCS	52	Applied GCS pre-operatively and IPC intra- and post-operatively until ambulation.	DVT: 5/104 (limbs)PE: 1	Hospital stay
		GCS	56	Applied GCS pre-operatively.	DVT: 14/112 (limbs)PE: 1	Hospital stay
**Nagata et al.Japan**	2015	IPC	14	Used IPC immediately prior to surgery, until full ambulation post operation.	DVT: 3PE: 3	9-11 days; Hospital stay
		LMWH	16	20mg enoxaparin initiated at 9:30 PM on postoperative day 2 and continued for 7days.	DVT: 1PE: 0	9-11 days; Hospital stay

*IPC: Intermittent Pneumatic Compression; LMWH: low molecular weight heparin; GCS: graduated compression stockings; DVT: deep vein thrombosis; PE: pulmonary embolism*.

**Table 2:** Study population and VTE measurements of included trials

Study	Inclusion criteria	Exclusion criteria	VTE measurements
**Clarke Pearson and Synan et al.1984**	Patients undergoing major surgery for confirmed or presumed gynecologic malignancies.	Those had received anticoagulants or with acute venous thromboembolic complications.	^125^I-fibrinogen counting and impedance plethysmography; suspicious DVT or PE was evaluated with venography, ventilation perfusion lung scanning or pulmonary arteriography.
**Clarke Pearson and Creasmann et al.1984**	Patients undergoing major surgery for known or presumed gynecologic malignancies.	Patients had VTE within 3 months or those had taken anticoagulants within 6 months.	^125^I-fibrinogen counting and impedance plethysmography; suspicious DVT or PE was evaluated with venography, ventilation perfusion lung scanning or pulmonary arteriography.
**Clarke-Pearson and Synan et al.1993**	Patients undergoing major surgery for known or presumed gynecologic malignancies.	A history of a bleeding diathesis, thromboembolism within 3 months, or receiving anticoagulation within 6 weeks	Impedance plethysmography, duplex Doppler ultrasonography, and ascending contrast venography. Further ventilation-perfusion lung scan and pulmonary arteriography for suspicious PE
**Maxwell et al.2001**	More than 40 years old, underwent major abdominal or pelvic surgeryfor diagnosed or suspected gynecologic malignancy.	DVT or PE within 6months; contraindications to heparin therapy; conduction anesthesia; history of heparin sensitivity; pregnancy; or history of coagulation abnormalities.	Real-time ultrasound compression technique with duplex and color Doppler imaging. Follow-up telephone to question patients regarding VTE signs and symptoms.
**Yang et al.2009**	Patients undergoing gynecological surgeries with high risk factor.	No specific description.	Ultrasonography examination of lower extremity.
**Gao et al.2012**	Patients undergoing gynecological pelvicsurgery with high-risk factors for DVT, aged more than 60 years old, a history of VTE, heart disease or varicose veins.	Thrombophlebitis; Acute DVT; Platelet count <100×10^9^/L or coagulopathy; spontaneous bleeding within six months; pulmonary edema etc.	Color Doppler flow imaging for DVT, and tomographic pulmonary angiography test if DVT was diagnosed.
**Nagata et al.2015**	over 40 years old and 40 kg weight, underwent major abdominal or pelvic surgery, with confirmed or suspected gynecologic malignancy	Preoperative confirmed VTE, hypersensitivity to heparin, severe liver or renal dysfunction, active bleeding etc.	Chest, abdominal, and lower extremities contrast-enhanced CT scan for DVT and PE.

VTE: venous thromboembolism prophylaxis. Other abbreviations as in Table 1.

### IPC use for DVT prophylaxis

**Figure 2:**
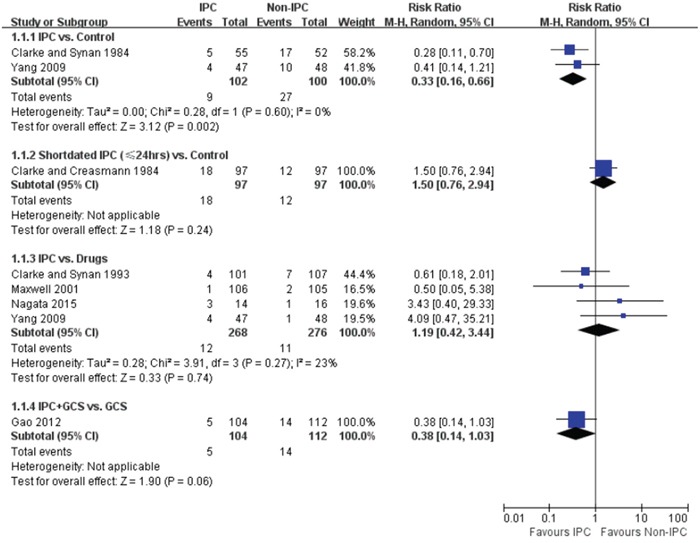
Forest plot of the effectiveness of IPC on DVT prophylaxis, stratified by IPC duration and comparator

All included studies reported the incidence of DVT. As shown in Figure 2, compared with control, routine IPC use significantly lowered the DVT risk (RR = 0.33, 95% CI: 0.16 – 0.66), however, short-dated (no more than 24 hours) use of IPC did not reduce the risk of DVT (RR = 1.5, 95% CI: 0.76 – 2.94). The incidence of DVT in IPC and drugs group was 4.5% (12 out of 268) and 3.99% (11 out of 276), respectively, and no significant between-group difference was observed (RR = 1.19, 95% CI: 0.42 – 3.44). According to the study by Gao et al. [17], which counted two lower limbs as two samples, the combination of IPC and GCS had a marginally lower risk of DVT than GCS alone (RR = 0.38, 95% CI: 0.14 – 1.03).

### IPC use for PE prophylaxis

**Figure 3:**
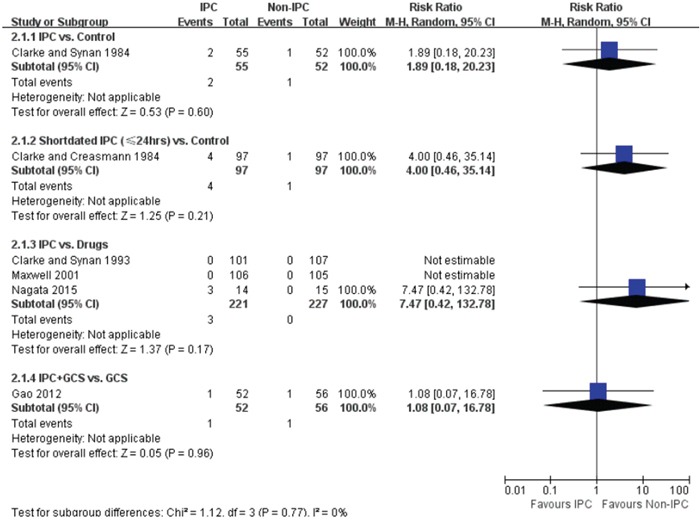
Forest plot of the effectiveness of IPC on PE prophylaxis, stratified by IPC duration and comparator

Six articles reported the rate of PE [12–15, 17, 18]. Two studies reported of no incidence of PE [14, 15]. As displayed in Figure 3, compared with control, neither routine (RR = 1.89, 95% CI 0.18 – 20.23) or short- dated (RR = 4.0, 95% CI: 0.46 – 35.14) use of IPC alter the risk of PE. Compared with drugs (0%, 0 out of 227), the frequency of PE was 1.4% (3 out of 221) in IPC group, however, no significant difference was found between IPC and drugs (RR = 7.47, 95% CI: 0.42 – 132.78). The rate of PE was similar in IPC plus GCS group (1 out of 52) and GCS alone group (1 out of 56) (RR = 1.08, 95% CI 0.07 – 16.78).

### IPC use for transfusion rate

**Figure 4:**
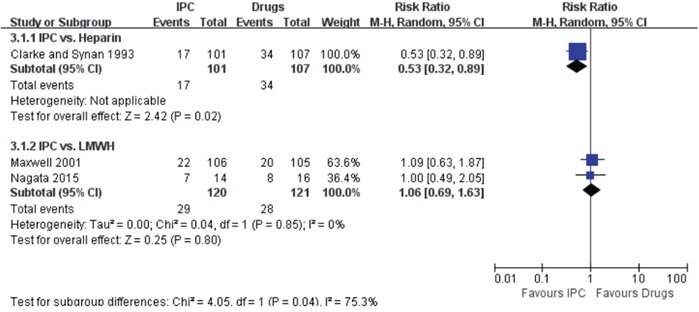
Forest plot of perioperative transfusion rate, stratified by comparator agent

Three trials mentioned perioperative transfusion rate in IPC and drugs group [14, 15, 18]. Compared with heparin, IPC was associated with a lower postoperative transfusion rate (16.8% in IPC vs. 31.8% in heparin, RR = 0.53, 95% CI: 0.32 – 0.89) (Figure 4). Compared with LWMH, patients treated with IPC had a similar transfusion rate in operating room (RR = 1.06, 95% CI: 0.69 – 1.63) (Figure 4).

### Sensitivity analysis

Sensitivity meta-analyses by applying fixed-effect model did not observe significant change in above results (Supplementary Figure 3).

## DISCUSSION

A majority of our study population are with gynecologic malignancies. Without VTE prophylaxis, reported postoperative incidence of DVT was as high as 37.9% in patients with gynecologic cancer [19]. Death occurs frequently in VTE cases, and approximately 12% of PE patients die within 30 days of diagnosis [1]. Therefore, prophylaxis for perioperative VTE is of great importance. Antithrombotic drugs are strongly recommended for patients with high risk of VTE, however, the nature of anticoagulants, including heparin and LWMH, may preclude clinicians from using these drugs timely. A major concern is the bleeding risk with anticoagulants. Anticoagulation may contribute to increased blood loss and transfusion rate during procedures. Furthermore, major bleeding in hospitalized surgical patients is an important predictor of mortality [20]. IPC is a popular mechanical thromboprophylaxis used in surgical patients. A previous meta-analysis demonstrated that IPC was effective in reducing VTE complications in hospitalized patients in comparison to control or thromboembolic deterrent stockings, with a lower risk of bleeding than pharmacological thromboprophylaxis [21]. However, that study included various hospitalized patients, including general surgical, critically ill and trauma patients. Hence the evidence might not be applicable to gynecological surgery patients. Recently, O'Connell et al. evaluated the use of IPC in orthopedic and neurosurgical postoperative patients, and found that IPC alone was neither superior nor inferior to pharmacological thromboprophylaxis [22]. Again, the evaluation of IPC use was not conducted in gynecological surgery subjects. Thus we performed this meta-analysis focusing on the use of IPC in patients undergoing gynecologic surgery.

In our analysis, we found that routine use of IPC (maintained for 5 days or until full ambulation) lowered DVT risk compared with control, but a short-dated IPC use (until discharge from the recovery room or 24 hours after operation) could not produce preventive effect. This suggests that IPC duration may affect the efficacy of IPC prophylaxis. Future studies may further explore the ideal IPC duration in gynecological surgery patients. Surprisingly, IPC and control had similar rate of PE, this may explained by the limited sample size, more large trials are needed.

Since IPC was effective in thromboprophylaxis, whether it is superior or inferior to chemoprophylaxis is of high interesting. In our pooled analysis, we found that IPC showed neither superiority nor inferiority to drugs in prevention for VTE, which is consistent with a previous meta-analysis in orthopedic and neurosurgical patients [22]. Furthermore, we observed that IPC had a lower rate of postoperative transfusion rate than heparin, and a similar rate of transfusion rate in operating room to LWMH. These findings indicate IPC or LWMH might be safer than heparin in gynecological surgery patients. However, the dose of heparin or LWMH may significantly influence the bleeding complications. As previously reported in gynecological surgery, compared with 5000 units heparin twice-daily, bleeding risk was higher in 5000 units LWMH daily but not in 2500 units LWMH daily [23, 24]. Due to the limited sample size, the difference in bleeding risk between IPC and drugs remains to be further investigated.

No studies but the one by Gao et al. evaluated the combined use of IPC and another thromboprophylaxis [17]. IPC plus another mechanical method –-GCS, seemed more effective than GCS alone. Sachdeva et al. advocated that GCS on a background of another prophylactic method was superior to the other method alone in DVT prophylaxis [25]. In this case, it is reasonable to compare the combination of IPC and chemoprophylaxis with IPC or chemoprophylaxis alone. Turpie et al. showed that IPC plus fondaparinux 2.5 mg reduced risk of VTE by approximately 70%, compared with IPC alone, in abdominal surgery [26]. Unfortunately, in patients undergoing gynecologic surgery, no specific randomized trials have investigated this issue.

### Limitations

Several limitations of our study should be noticed. First, included studies were rather heterogeneous in many respects. Follow up time ranged from hospital stay to 42 days, drug regimens were various, comparator was control, drugs or GCS. Especially, as shown in Table 2, there are variations in VTE measurements and diagnostic strategy, which may affect observed incidences of thromboembolism. However, these measurements were all validated in clinical practice, and both symptomatic and “silent” VTE (a combination of the two) are all relevant to patients’ health. Second, the small number of included studies may produce publication bias, which has been estimated by the funnel plot. However, the test power of funnel plots becomes low in such a meta-analysis involving 7 studies. Last, it is difficult to achieve blinding of participants in such trials, and thus performance bias cannot be excluded.

In conclusion, IPC is effective in reducing DVT complications in gynecologic surgery. IPC is neither superior nor inferior to pharmacological thromboprophylaxis, but might be safer than heparin in patients undergoing gynecologic surgery. However, whether combination of IPC and chemoprophylaxis is more effective than IPC or chemoprophylaxis alone remains unknown in these patients.

## MATERIALS AND METHODS

We performed this systematic review and meta-analysis in accordance with the PRISMA guidelines [27].

### Data sources and literature search

A computerized literature search was conducted in Pubmed, Embase and Cochrane Library databases by two investigators (YTX and JPF) independently, from inception through July 2016. The following searching terms were used: pneumatic compression, sequential compression, external compression, intermittent compression, venous thromboembolism, deep vein thrombosis, pulmonary embolism, gynecological and gynecology. No language restriction was applied. We also searched potentially eligible articles in the reference lists of retrieved records.

### Study selection

Any RCT that evaluated the use of IPC in gynecologic surgery was included. Namely, RCTs that compared IPC with control or drugs, and those compared a treatment plus IPC with that treatment alone were all included. Observational studies and review articles were excluded. Studies that did not report outcomes of interest were excluded either. Titles and/or abstracts were screened by two separate investigators (JPF and ZQF). After removing obviously irrelevant articles, remained full texts were further evaluated for eligibility. Any disagreement was resolved by a third party (ZJG).

### Data extraction and quality assessment

Two reviewers (YTX and JPF) separately extracted data of finally identified articles, including study population, publication year, follow up duration, treatment regimen, occurrence of VTE complications (i.e. DVT and/or PE), perioperative transfusion rate and outcome measurements. The frequencies of VTE and transfusion rate were taken as efficacy and safety endpoints, respectively. The quality assessment of included studies was conducted by the mean of Cochrane Collaboration Risk of Bias Tool. (Supplementary Figure 1)

### Data synthesis and statistical analysis

Mantel–Haenszel method was applied to pool data on efficacy and safety outcomes, with a random-effect model. The overall effect estimates of the outcomes were all reported as risk ratios (RR) and 95% confidence interval (CI). The heterogeneity between included studies was estimated using I^2^ test. We considered heterogeneity significant if I^2^ value was more than 50%. Fixed-effect model was also applied as the sensitivity analysis if I^2^value was smaller than 50%. Publication bias was assessed by the funnel plot. Necessary subgroup analyses were also performed, stratified by comparator or treatment regimen. The software Review manager (version 5.2) provided by Cochrane Collaboration was used for all statistical analysis.

## SUPPLEMENTARY FIGURES


