# Supplementary material for: Efficacy of intermittent pneumatic compression for venous thromboembolism prophylaxis in patients undergoing gynecologic surgery: A systematic review and meta-analysis
Source: Oncotarget. 2016 Nov 25;8(12):20371–9. doi: 10.18632/oncotarget.13620 (PMC5386769; doi:10.18632/oncotarget.13620)
Supplement: Supplementary file 1 [file oncotarget-08-20371-s001.pdf]

Efficacy of intermittent pneumatic compression for venous thromboembolism prophylaxis in patients undergoing gynecologic surgery: A systematic review and meta-analysis

SUPPLEMENTARY FIGURES

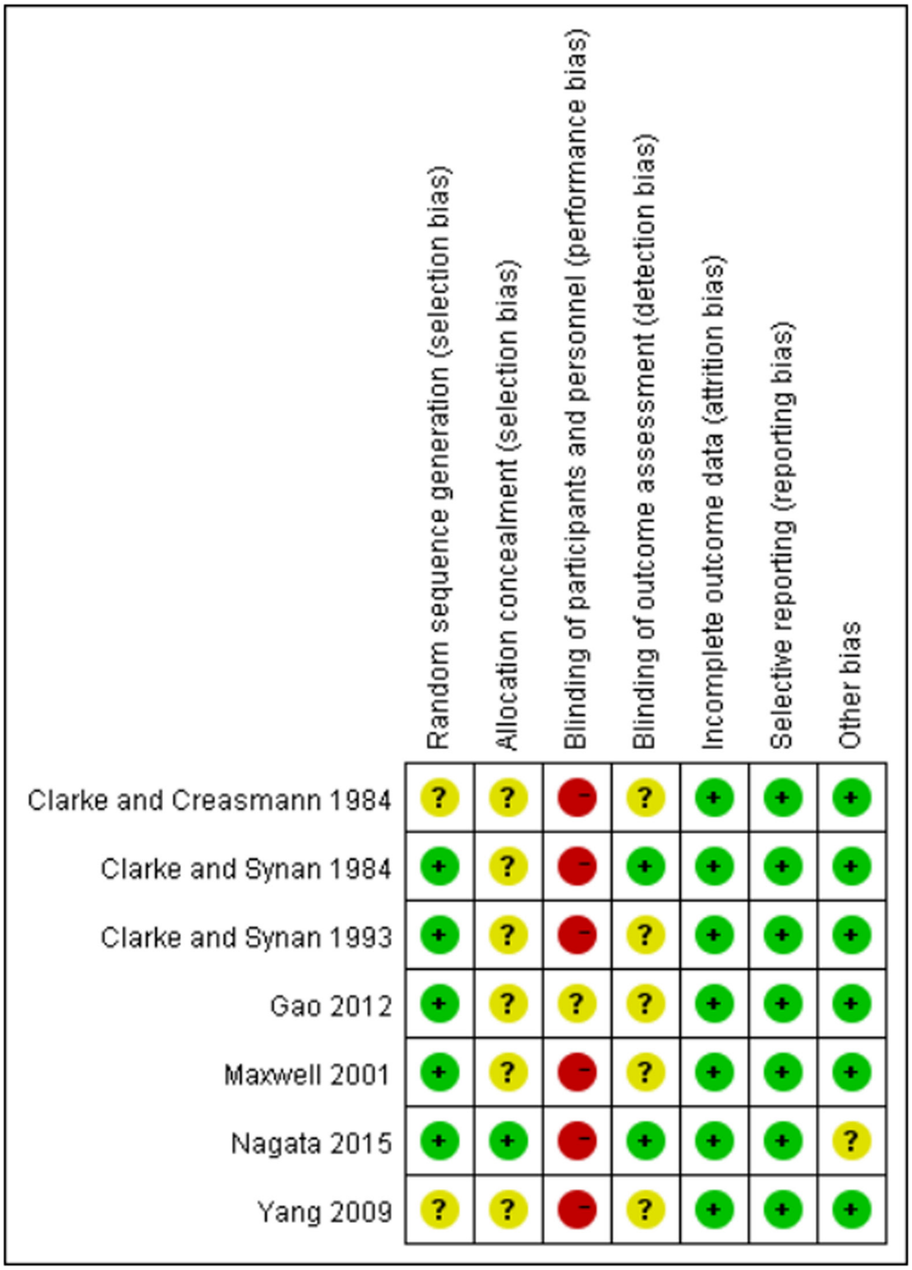

Supplementary Figure 1: Risk of bias summary: review authors' judgements about each risk of bias item for each included study.

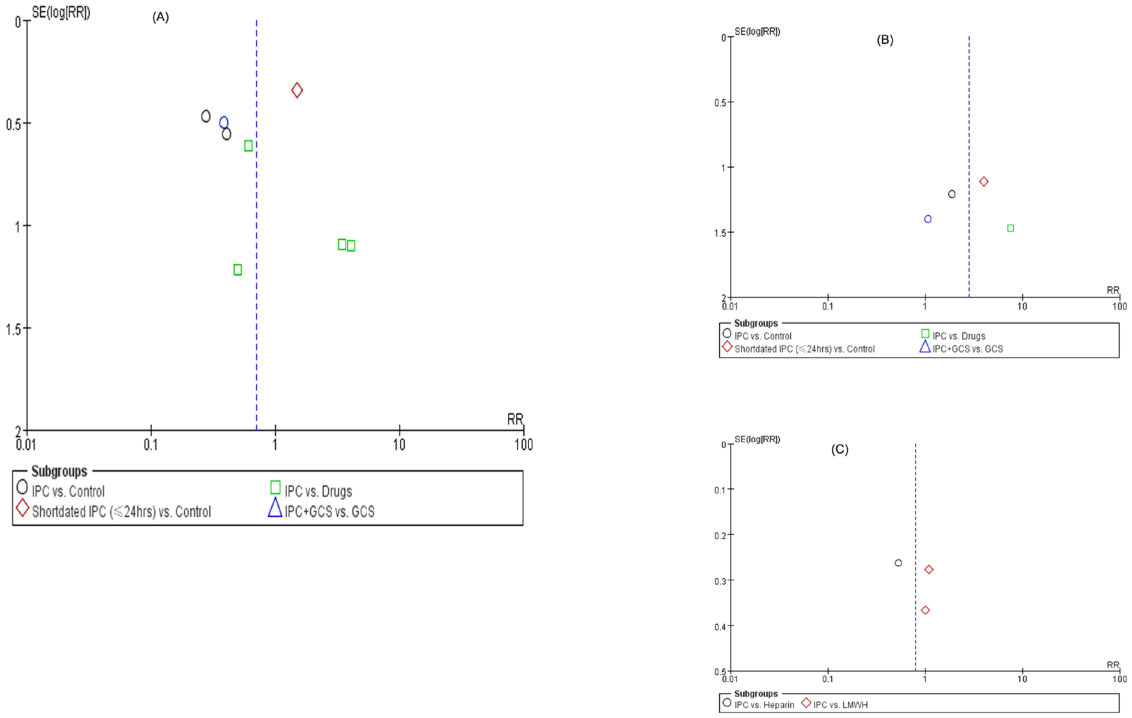

Supplementary Figure 2: Funnel plots of A. DVT, B. PE, and C. transfusion rates.

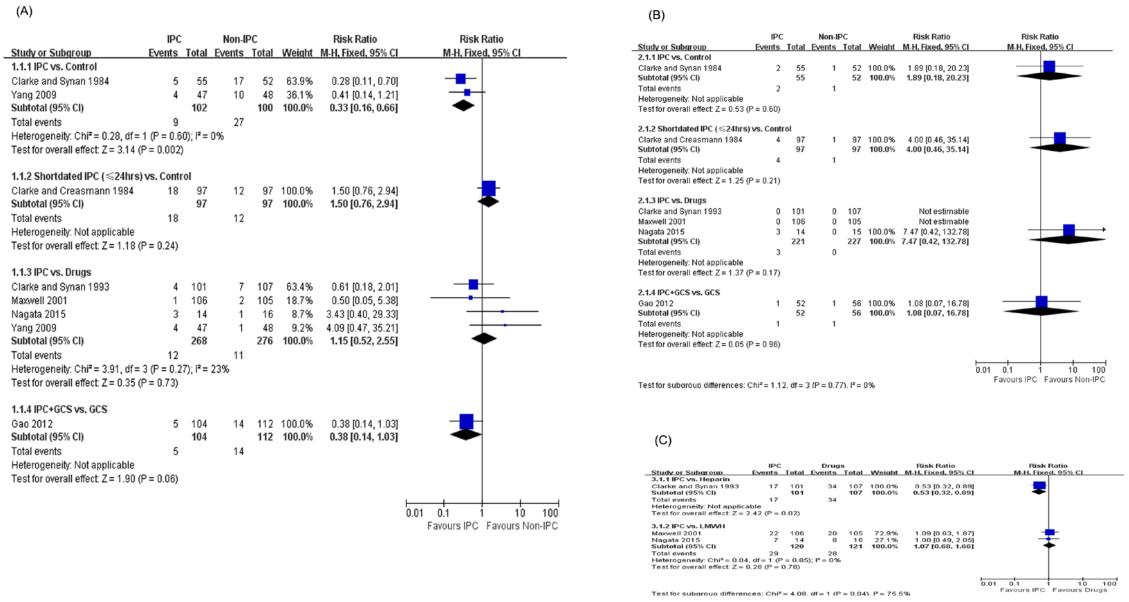

Supplementary Figure 3: Fixed-effect model meta-analysis of A. the effectiveness of IPC on DVT prophylaxis, stratified by IPC duration and comparator, B. the effectiveness of IPC on PE prophylaxis, stratified by IPC duration and comparator, and C. perioperative transfusion rate, stratified by comparator agent.
